# Health-care associated infections rates, length of stay, and bacterial resistance in an intensive care unit of Morocco: Findings of the International Nosocomial Infection Control Consortium (INICC)

**DOI:** 10.1186/1755-7682-2-29

**Published:** 2009-10-07

**Authors:** Naoufel Madani, Victor D Rosenthal, Tarek Dendane, Khalid Abidi, Amine Ali Zeggwagh, Redouane Abouqal

**Affiliations:** 1 Ibn Sina Hospital- Medical ICU, Rabat, Morocco; 2 Medical College of Buenos Aires, Buenos Aires, Argentina; 3 Laboratory of Biostatistics, Clincial and Epidemiological Research, Faculté de Médecine et de Pharmacie, Rabat, Morocco

## Abstract

**Background:**

Most studies related to healthcare-associated infection (HAI) were conducted in the developed countries. We sought to determine healthcare-associated infection rates, microbiological profile, bacterial resistance, length of stay (LOS), and extra mortality in one ICU of a hospital member of the International Infection Control Consortium (INICC) in Morocco.

**Methods:**

We conducted prospective surveillance from 11/2004 to 4/2008 of HAI and determined monthly rates of central vascular catheter-associated bloodstream infection (CVC-BSI), catheter-associated urinary tract infection (CAUTI) and ventilator-associated pneumonia (VAP). CDC-NNIS definitions were applied. device-utilization rates were calculated by dividing the total number of device-days by the total number of patient-days. Rates of VAP, CVC-BSI, and CAUTI per 1000 Device-days were calculated by dividing the total number of HAI by the total number of specific Device-days and multiplying the result by 1000.

**Results:**

1,731 patients hospitalized for 11,297 days acquired 251 HAIs, an overall rate of 14.5%, and 22.22 HAIs per 1,000 ICU-days. The central venous catheter-related bloodstream infections (CVC-BSI) rate found was 15.7 per 1000 catheter-days; the ventilator-associated pneumonia (VAP) rate found was 43.2 per 1,000 ventilator-days; and the catheter-associated urinary tract infections (CAUTI) rate found was 11.7 per 1,000 catheter-days.

Overall 25.5% of all *Staphylococcus aureus *HAIs were caused by methicillin-resistant strains, 78.3% of *Coagulase-negative-staphylococci *were methicillin resistant as well. 75.0% of *Klebsiella *were resistant to ceftriaxone and 69.5% to ceftazidime. 31.9% of *E. Coli *were resistant to ceftriaxone and 21.7% to ceftazidime. 68.4% of *Enterobacter sp *were resistant to ceftriaxone, 55.6% to ceftazidime, and 10% to imipenem; 35.6% of *Pseudomonas sp *were resistant to ceftazidime and 13.5% to imipenem.

LOS of patients was 5.1 days for those without HAI, 9.0 days for those with CVC-BSI, 10.6 days for those with VAP, and 13.7 days for those with CAUTI.

Extra mortality was 56.7% (RR, 3.28; P =< 0.001) for VAP, 75.1% (RR, 4.02; P = 0.0027) for CVC-BSI, and 18.7% (RR, 1.75; P = 0.0218) for CAUTI.

**Conclusion:**

HAI rates, LOS, mortality, and bacterial resistance were high. Even if data may not reflect accurately the clinical setting of the country, programs including surveillance, infection control, and antibiotic policy are a priority in Morocco.

## Background

The industrialized countries have established standardized criteria for the devise of institutional healthcare-associated infection (HAI) surveillance and infection control (IC) measures [1]. Most studies related to HAI were conducted in the developed countries [2-4] and demonstrated the efficacy of HAI surveillance [3-5] and its significant incidence concerning patient morbidity and mortality [6].

Conversely, in the developing countries, few studies provide data of device associated infection rates using the standardized definitions HAI rates per 1000 device days[7-11]. In Morocco, there is one published study on prevalence of HAI conducted in a university hospital, which showed a significantly high HAI rate [12].

This study presents data collected by the INICC that show HAI in a Moroccan ICU with the aim of benchmarking them with regional and international standards and assess the need for further specific infection control interventions.

## Methods

### Setting

A prospective study was performed of all patients consecutively admitted to a 12-bed medical ICU of Rabat University Hospital between November 2004 and April 2008. Rabat University Hospital is the referral venue for habitants in western-north Morocco. The 12-bed medical ICU admits approximately 550 patients annually with an average age of 40 years. Surgery patients, coronary patients, neonates and burn patients are treated in specialized units.

The hospital has an infection control team (ICT) with a physician and an infection control practitioner (ICP) with 16 years of experience (Table 1) and a microbiology laboratory to provide *in vitro *susceptibility testing of clinical isolates using standardized methods.

**Table 1:** Patients characteristics

Surveillance Period	11/04 to 04/08
Experience of the infection control practitioner, *y*	16
Patients studied, *n*	1.731
Total ICU days, *d*	11.297
Men, %	52.4
Mean age, *y*, ± SD	42.4 ± 19.2
Mean ASIS, ± SD	3.9 ± 0.7
Mean APACHE II, ± SD	11.5 ± 8.2
Device use	
Ventilator days, *d*	3.2
Ventilator use, proportion	0.28
CVC days, *d*	2.2
CVC use, proportion	0.19
Urinary catheter days, d	6.8
Urinary catheter use, proportion	0.60

ASIS = average severity of illness score; CVC = central venous catheter; ICU = intensive care unit.

The hospital's institutional review board agreed to the study protocol, and patient confidentiality was protected by codifying the recorded information making it only identifiable to the ICT. Informed consent was not demanded because this observational study did not require any deviation from routine medical practice.

### Surveillance

Rates of central vascular catheter-associated bloodstream infection (CVC-BSI), catheter-associated urinary tract infection (CAUTI) and ventilator-associated pneumonia (VAP) were determined monthly, and CDC-NNIS definitions were applied [13,14].

### Crude Excess Mortality

Crude excess mortality is the difference between the crude overall case-fatality of patients with HAI and the crude case-fatality of patients without HAI in the ICU during the same period.

Length of stay (LOS) was collected prospectively when filling INICC forms daily. Adult patients with HAI were considered cases, while those without HAI were considered controls. We calculated extra-LOS subtracting average LOS of patients with and without HAI.

### Training, Forms, Validation, and Data Feedback

The INICC chairman (Victor D Rosenthal) provided training procedures related to surveillance to the participating hospital, which daily filled forms including data related to patient, such as demographics, age, gender, severity of illness score, and hospital location. These data were gathered at admission to the ICU. After admission, data on mechanical ventilation (MV), placement of central vascular catheter (CVC) and urinary catheters (UC), fever, blood pressure, antibiotic use, and the results of cultures on patients hospitalized in the ICU were collected. Upon the end of hospitalization, the ICP registered data regarding patients with HAI that included the date of onset, site of HAI, infecting microorganisms and their antibiotic susceptibilities.

The average severity of illness score (ASIS) was recorded by using the CDC-NNIS criteria [14].

Patients in the ICU had a check-off when the ICP in charge of reviewing the filled forms was satisfied that the clinical and microbiologic criteria for the specific type of HAI had been met. The ICT in Morocco had access to a team at the central office (CO) in Buenos Aires, which provided responses checked by the chairman to inquiries within 24 hours.

Filled out forms were sent monthly from each ICT to the CO, where an HAI adjudication process of each case was performed by analyzing the recorded signs (Fever, Blood pressure) and cultures, in order to assure that the CDC NNIS criteria for HAI were met [13,14]. Lastly, the forms were further uploaded in the database. The CO team prepared and sent to the ICT monthly reports, showing global rates per 100 patients and per 1000 bed-days, HAI per 1000 device-days, microbiological profile, extra mortality by type of HAI, extra-LOS, hand hygiene compliance (HHC), and CVC and UC care compliance.

The HHC was observed and monitored before patient contact by a randomized evaluation, 3 times a week during one hour each time, during all working shifts (morning, afternoon, night) and for all health care workers (physicians, nurses, ancillary staff).

### Culture Techniques

#### VAP

In most cases, a deep tracheal aspirate from the endotracheal tube was cultured aerobically and gram-stained.

#### CVC-BSI

CVC were removed aseptically and the distal 5 cm of CVC was amputated and cultured using a standardized semiquantitative method [15]. Concomitant blood cultures were drawn percutaneously in nearly all cases.

#### CAUTI

A urine sample was aseptically aspirated from the sampling port of UC and cultured quantitatively. In all cases, standard laboratory methods were used to identify microorganisms, and a standardized susceptibility test was performed [16].

### Definitions

#### Ventilator-Associated Pneumonia

Ventilator-associated pneumonia is indicated in a mechanically ventilated patient with a chest radiograph that shows new or progressive infiltrates, consolidation, cavitation, or pleural effusion. The patient must also have at least 1 of the following criteria: new onset of purulent sputum or change in character of sputum; organism cultured from blood; or isolation of an etiologic agent from a specimen obtained by tracheal aspirate, bronchial brushing or bronchoalveolar lavage, or biopsy.

#### Laboratory-Confirmed CVC-Associated Bloodstream Infection

Central venous catheter-associated bloodstream infection is laboratory-confirmed when a patient with a CVC has a recognized pathogen that is isolated from 1 or more percutaneous blood cultures after 48 hours of vascular catheterization and is not related to an infection at another site. The patient also has at least 1 of the following signs or symptoms: fever (temperature > 38°C), chills, or hypotension. With skin commensals (for example, diphtheroids, *Bacillus *spp., *Propionibacterium *spp., *Coagulase-negative staphylococci*, or micrococci), the organism is cultured from 2 or more blood cultures.

#### Catheter-Associated Urinary Tract Infection

For the diagnosis of catheter-associated urinary tract infection, the patient must meet 1 of 2 criteria. The first criterion is when a patient with a urinary catheter has 1 or more of the following symptoms with no other recognized cause: fever (temperature > 38°C), urgency, or suprapubic tenderness when the urine culture is positive for 10^5 ^colony-forming units per mL or more, with no more than 2 microorganisms isolated. The second criterion is when a patient with a urinary catheter has at least 2 of the following criteria with no other recognized cause: positive dipstick analysis for leukocyte esterase or nitrate, pyuria (≥ 10 leukocytes per mL of urine), organisms seen on gram stain, physician diagnosis of urinary tract infection, or physician initiates appropriate therapy for a urinary tract infection.

### Statistical analysis

EpiInfo^® ^version 6.04b (CDC, Atlanta, Ga) was used for data analysis. Device-utilization rates were calculated by dividing the total number of device-days by the total number of patient-days. Rates of VAP, CVC-BSI, and CAUTI per 1000 device-days were calculated by dividing the total number of HAI by the total number of specific device-days and multiplying the result by 1000 [14].

## Results

### Features of Population Studied

During the three years and 6 months of study in one Moroccan ICU surveillance data were prospectively collected on 1,731 patients hospitalized in the ICU for 11,297 ICU-days (Table 1). They acquired 251 HAIs, an overall rate of 14.5% or 22.2 HAIs per 1,000 ICU-days. VAP represented 54.6% of all HAIs. CAUTI represented 31.9%. CVC-BSI represented 13.5% (Table 2). Individual characteristics of each ICU, the number of patients enrolled in the study, the number of ICU-days, APACHE II score and ASIS are shown in Table 1. Mean patient ASIS, being 3.88 overall, and APACHE II 11.5.

**Table 2:** HAIs per 1000 devices days: VAP, CVC-BSI, and CAUTI.

***Infection site***	***Device type***	***Device-days***	***Device utilization***	***HAI***	***Distribution of HAI (%)***	***Rate per 100 patients***	***Rate per 1000 device-days***
VAP	MV	3.174	0.28	137	54.6%	7.9%	43.16
CVC-BSI	CVC	2.164	0.19	34	13.5%	2.0%	15.71
CAUTI	UC	6,814	0.60	80	31.9%	4.6%	11.74

HAI: Health care associated infection; VAP: Ventilator-associated pneumonia, CVC-BSI: central vascular catheter-associated blood stream infection. CAUTI: catheter-associated urinary tract infection; MV: Mechanical Ventilator; UC: Urinary catheter.

### Device-Utilization Ratio

The ICU device utilization was as follows: for MV, 0.28; for CVC, 0.19; and for UC, 0.60. Distributions by type of HAI and device-utilization are shown in Table 2.

### VAP

The overall rate of VAP in the ICU was 43.16 per 1,000 MV days (Table 2). Crude mortality of patients with VAP was 81.6%, with extra mortality of 56.7%, (RR 3.28, 95% CI 2.51-4.29, P < 0.001). Patients without HAI presented a crude mortality rate of 24.9%, yielding an excess mortality of 56.7%. LOS of patients without HAI was 5.1 days and of patients with VAP, 10.6 days (RR, 2.08; 95% CI, 1.94 - 2.24; P < 0.0001), representing 5.5 extra days.

### CVC-BSI

The overall rate of CVC-BSI in the ICU was 15.71 per 1,000 CVC days (Table 2). The crude mortality of patients with CVC-BSI was 100%, with extra mortality of 75.1% (RR 4.02, 95% CI 1.50 - 10.77, P = 0.0027). LOS of patients with CVC-BSI was 9.0 days (RR, 1.76; 95% CI, 1.27 - 2.45; P = 0.0005), representing 3.9 extra days.

### CAUTI

The overall rate of CAUTI in the ICU was 11.7 per 1,000 urinary catheter days (Table 2). Crude mortality of patients with CAUTI was 43.6%, with extra mortality for CAUTI of 18.7% (RR 1.75, 95% CI 1.08-2.85, P = 0.0218). LOS of patients with CAUTI was 13.7 days (RR, 269; 95% CI, 2.47 - 2.94; P < 0.0001), representing 8.6 extra days.

### Use of Antibiotics, Overall Bacterial Profile and Resistance

The use of antibiotics per 1000 ICU days is shown in Table 3. Overall 24.1% of all HAI were caused by *Acinetobacter sp*. --78.6% of which were resistant to piperaciline tazobactam; 23.1% was caused by *Pseudomonas sp *infections --35.6% were resistant to ceftazidime and 13.5% to imipenem; 15.4% were caused by *Klebsiella sp*. --75.0% of which were resistant to ceftriaxone, 69.5% to ceftazidime; 12.8% were caused by *E. Coli *--31.9% of which were resistant to ceftriaxone, 21.7% to ceftazidime; 8.2% were caused by *Candida sp; *4.1% by *Enterobacter sp *--68.4% of which were resistant to ceftriaxone, 55.6% to ceftazidime, and 10% to imipenem; 4.6% were caused by *S aureus *infections --25.5% of which were resistant to methicilin; 3.1% by *Coagulase-negative-staphylococci *--78.3% of which were resistant to methicilin; 2.6% by *Proteus sp*; 1.0% were caused by *Streptocococcus sp; *1.0% by *Serratia sp*.

**Table 3:** Use of Antibiotics per 1,000 ICU-days

**Antimicrobial class**	**No of defined daily doses**	**Pooled mean**
Penicillin group	269	23.81
Ampicillin Group	2290	202.71
Antipseudomonal penicillins	79	6.99
Antistaphylococcal penicillins	3	0.27
First-generation cephalosporins	3	0.27
Second-generation cephalosporins	85	7.52
Third-generation cephalosporins	2435	215.54
Carbapenems	355	31.42
Fluoroquinolones	1204	106.58
Trimethoprim-sulfamethoxazole	235	20.80
Vancomycin	197	17.44

### Hand washing compliance

We observed 5,928 patient contacts. The overall HHC rate was 32.1%. The results stratified by working shifts, type of contact, and health care workers are shown in Table 4.

**Table 4:** Hand Hygiene per Stratum

	**Hand** **Hygiene %**	**n° of observations**	**Comparisons**	**RR**	**95%CI**	**P. value**
Overall Hand Hygiene	32.1%	5.928	-			
Strafied:						
Physicians	59.0%	1.684	Ph vs. Nurses	2.63	2.40-2.89	0.0001
Nurses	22.4%	3.824	Nurses vs. Anc Staff	1.74	1.32-2.29	0.0006
Ancillary Staff	12.9%	420	Ph. Vs Anc Staff	4.59	3.49-6.04	0.0001
Men	38.2%	2.370	Men vs Women	1.36	1.24-1.49	0.0001
Women	28.1%	3.558	-			
Non invasive contact	26.5%	2.902	Invasive vs non-invasive	1.41	1.29-1.55	0.0001
Invasive contact	37.5%	3.026	-			
Morning Shift	38.2%	2.016	Morning vs Afternoon	1.00	0.91-1.11	0.9696
Afternoon Shift	38.1%	2.054	Morning vs Night	2.02	1.78-2.29	0.0001
Night Shift	18.9%	1.858	Night vs Afternoon	2.01	1.77-2.28	0.0001

## Discussion

This study is the first to show HAI rates per 1000 device-days, bacterial resistance, mortality, and extra LOS in one Moroccan city. HAI, which can be reduced by 30% applying targeted device-associated surveillance of HAIs according to studies from the Europe and USA, [3,5] increases healthcare costs and mortality [17-19]. Surveillance forms were devised by the INICC --founded in 1998 with Latin American hospitals [8-10,17,20] for data collection of patients with and without HAI, which enabled the ICP to match features and determine extra LOS, costs, mortality [19,20] and major HAI risk factors.

This study showed a lower use of MV (0.28 vs. 0.45), CVC (0.19 vs. 0.59) and UC (0.60 vs. 0.76) as compared to the device utilization reported by the US in the National Healthcare Safety Network (NHSN) [21]. HAI distribution was: VAP (54.6%), CVC-BSI (13.5%), and CAUTI was (31.9%), which is similar with the INICC overall data, where VAP represented 41.0% of all HAIs, followed by CVC-BSI (30.0%) and CAUTI (29.0%) [7].

Our overall HAI rate per 100 patients, being 14.5%, was similar than the 14.7% rate of a previous INICC study [7]. The rate of HAI per 1,000 bed-days was 22.2, slightly lower than the overall INICC rate (22.5/1000) [7].

Our CVC-BSI overall rate was 15.7 per 1000 CVC-days, which is higher than the INICC overall rate of 12.5, [7] and much higher than the NHSN 2.9 rate [21]. Our overall VAP rate by 1,000 MV-days was 43.2, higher than the 24.1 INICC rate [7] and than the NHSN 3.1 rate [21]. Our CAUTI rate (11.7 per 1000 UC days) was also higher than the INICC (8.9) [7] and NHSN (4.4) (table 5) [21].

**Table 5:** Comparison of device associated infection rates (per 1000 device-days) in the studied Moroccan ICU, in ICUs of the International Nosocomial Infection Control Consortium (INICC) and the U.S. National Healthcare Safety Network (NHSN).

	**Studied ICU** **(Morocco)** **2004-2008** **Pooled Mean**	**INICC** **2002-2005** **Pooled Mean**	**U.S. NHSN 2005-2006 Pooled Mean (Interquartile range, 25%-75%)**
Medical ICU			
CVC-BSI	15.7	12.5	2.9 (0.8-4.2)
CAUTI	11.7	8.9	4.4 (1.8-5.6)
VAP	43.2	24.1	3.1 (0.9-4.6)

CVC-BSI: central vascular catheter-associated blood stream infection, CAUTI: catheter-associated urinary tract infection, VAP: Ventilator-associated pneumonia.

Extra mortality of patients with VAP was 56.7%, much higher than the 27.8% found in the INICC study; [7] extra mortality of patients with CVC-BSI was 75.1%, higher than the 18.0% of the INICC; and extra mortality of patients with CA-UTI was 18.7%, which is slightly lower than the 21.3% found in the INICC study; [7] all them were significantly higher than the mortality of patients without HAI.

25.5% of *S aureus *were MRSA which is lower than the resistance of 52.9% presented by the NNIS, [4] and much lower than the 84% rate found in the INICC global study [7]. We also found a 78.3% *Coagulasa negative staphylococci *resistance to methicilin, which is slightly higher than the NNIS rate (76.6%) [4].

Regarding the resistance of *Pseudomonas sp *to imipenem, our 13.5% resistance rate was lower than the NNIS (19.1%) but our ceftazidime resistance of 35.6% was higher (35.6% vs 13.9%) [4].

This ICU from Morocco show high HAI rates, the reasons for this were explained in studies from developing countries, [12,22] which do not have a legal framework regarding IC programs or their implementation is poor, and have restricted funds [23,24], low nurse-to-patient staffing ratios, over-crowded wards, insufficient supplies contributing to high HAI rates, as stated in studies from US [25]. Also HHC rate is highly variable. In a previous INICC study, the overall HHC was 51% [26]. In the 2007 study, the HHC ranged between 49% and 69%.

HAI rates can be reduced by applying HAI surveillance [3,5], and simple effective IC practices. INICC evidenced that HHC substantially increased by the institution of programs, and there was a reduction in the CVC-BSI, CAUTI and VAP incidence at INICC members [27-33].

Some of our limitations are that our data do not reflect an entire country, but were collected prospectively over 4 years of comprehensive surveillance in one ICU at a representative city of Morocco.

HAI, being a threat to patient safety, requires improvement in clinical practice, by implementing HAI surveillance, and effective infection control interventions.

## Conclusion

HAI rates, LOS, mortality, and bacterial resistance were high in this ICU. Programs including surveillance, infection control, and antibiotic policy are a priority in Morocco.

## Competing interests

The authors declare that they have no competing interests.

## Authors' contributions

NM participated in the design of the study, and helped to draft the manuscript. VDR conceived of the study, and participated in its design and performed the statistical analysis and draft the manuscript. TD, KA and AAZ supervised research assistants in abstracting data from patients' case note and participated in the interpretation of the findings. RA analyzed the data and participated in the interpretation of the findings and drafting of the manuscript. All authors read and approved the final manuscript.

## Authors 'information

VDR is the International Infection Control Consortium's chairman.
